# Community knowledge, attitudes and practices on Yellow fever in South Omo area, Southern Ethiopia

**DOI:** 10.1371/journal.pntd.0006409

**Published:** 2018-04-09

**Authors:** Mengistu Legesse, Adugna Endale, Woldearegay Erku, Getachew Tilahun, Girmay Medhin

**Affiliations:** 1 Aklilu Lemma Institute of Pathobiology, Addis Ababa University, Addis Ababa, Ethiopia; 2 College of Health Sciences, School of Medicine, Department of Microbiology, Immunology & Parasitology, Addis Ababa University, Addis Ababa, Ethiopia; Institute for Disease Modeling, UNITED STATES

## Abstract

**Background:**

Yellow fever (Yf) outbreak was recently reported in South Omo of Southern Ethiopia. This area was also highly affected by Yf outbreak in the 1960s. However, there is no reliable information on the level of community knowledge attitudes and practices about the disease in the area. The objective of the current study was to assess level of community knowledge, attitudes and practices about Yf.

**Methods:**

Between March and May 2017, a community-based cross-sectional survey was conducted in two districts of the South Omo area. During the survey, 612 randomly selected adults were interviewed about Yf using structured questionnaire.

**Results:**

Out of the 612 study participants, 508 (83.0%) reported that they heard about Yf which is locally known as “a disease that causes vomiting blood”. Most (90.4%) of the study participants also said that Yf is different from malaria. Two hundred thirteen (41.9%) participants said that Yf can be transmitted from a patient to another person, while only 80 (37.6%) mentioned that the disease is transmitted through mosquitoes bite. Out of 333 (65.7%) study participants who believed that Yf is a preventable disease, 280 (84.1%) mentioned vaccine as a preventive method. The majority believed that the disease is a killer (97.2%) and a newly emerging (69.4%). Among the total of 612 study participants, 221(36.1%) were considered as having a high level of overall knowledge of Yf. Having educational level above 7^th^ grade (AOR = 3.25, 95% CI: 1.39, 7.57, p = 0.006) and being resident of Bena-Tsemay district (AOR = 1.77, 95% CI: 1.12, 2.78, P = 0.014) were significantly associated with having a high level of overall knowledge of Yf. Agro-pastoralism as an occupation compared to farming was associated with having a low level of overall knowledge of Yf (AOR = 0.51, 95% CI, 0.33, 0.79, P = 0.003).

**Conclusion:**

The findings indicate that most of the study community members had a low level of overall knowledge of Yf, especially about its cause, mode of transmission and preventive methods. Thus, there is a need to increase people’s knowledge and practices regarding the cause, mode of transmission and preventive methods like avoiding mosquitoe breeding sites beside vaccination through various strategies like disseminating information through community health extension workers and community leaders in the study area.

## Author summary

Yellow fever is becoming one of the most important re-emerging mosquito-borne viral diseases in many African countries despite the availability of an effective vaccine. Hence, assessing information on what a community knows about Yellow fever would contribute to the design of appropriate control strategies in addition to increasing access for vaccine. In this study, we assessed knowledge, attitudes and practices of local community about Yellow fever in South Omo area, southern Ethiopia, where outbreaks have occurred repeatedly since the 1960s. We found that the study community members had low knowledge about the cause and mode of transmission of the disease though they knew that it is a killer and affects all age groups. More than half of the study participants believed that the disease can be transmitted from a patient to another person through breathing. In the present study area, providing information to community members through community health extension workers regarding the role of mosquitoes in the transmission of this disease, and teaching what to do to minimize mosquitoes bite in understandable way would be helpful to increase their awareness about Yellow fever.

## Introduction

Yellow fever (Yf) remains a major public health problem in Africa since the 1930s, especially in the endemic areas of equatorial rain forest, the moist savanna and the dry savanna areas [1], despite the availability of effective vaccine for this disease. Previously, it was estimated that Yf causes 200,000 cases and 30,000 deaths annually, of which over 90% occurred in Africa [1]. According to a recent estimate, there were 130,000 Yf cases and 78,000 deaths in Africa for the year 2013 [2]. The disease is considered as one of the common re-emerging diseases in South America and many African countries like Democratic Republic of Congo, Sudan, Cameroon, Chad, Senegal, Côte d’Ivoire, Uganda, Sierra Leone, Ethiopia and Angola, where there is a low vaccine coverage or vaccine had waned [2–5]. In Ethiopia, large and small outbreaks of Yf occurred repeatedly since the 1960s and more recently, between November 2012 and October 2013 Yf outbreak occurred and resulted in many cases and deaths in South Omo area, southern Ethiopia, the same area which was highly affected by the outbreak in the 1960s [6–8].

The virus is transmitted to humans through the bite of widely distributed different species of the *Aedes* and *Haemagogus* mosquitoes. Thus, studies suggest that all areas in Africa where environmental conditions are suitable for mosquitoes breeding can be considered as areas at high risk of transmission; and the resurgence of Yf will continue unless vaccination is supported by effective mosquitoes control [9,10]. Moreover, it is suggested that control of mosquito-borne diseases like Yf requires effective participation of the local community [11]. Hence, assessing information on what a community knows about Yf would contribute to the efforts to design appropriate control strategies in addition to increasing access for vaccine. In this study, knowledge, attitudes and practices of local community about Yf was assessed in South Omo area, Southern Ethiopia, where Yf outbreak was recently reported.

## Methods

### Ethics statement

The study protocol was approved by the Institutional Review Board (IRB) of the Aklilu Lemma Institute of Pathobiology (ALIPB), Addis Ababa University. The aim of the study was explained to each of the participant and verbal consent was obtained because most of the study participants were illiterate. Each participant was interviewed independently and the collected information was kept confidential.

### Study area and population

The study was conducted in South Omo Zone, one of the 13 zones in Southern Nations, Nationalities and Peoples' Region (SNNPR). The Zone is located at about 750 km to the south of Addis Ababa and borders with Kenya on the south, a country which reported repeated outbreaks of arboviruses. The zone has eight districts with a total population of 573,435, and the majority of the inhabitants are practicing agro-pastoralism. Detailed information on the study Zone and the study population has been described elsewhere [12].

Among the six districts where Yf outbreak occurred between 2012 and 2013, two adjacent districts (Bena Tsemay and Debub Ari) were purposely selected for the present study based on the reported number of cases and deaths from the two areas [8]. Debub Ari district is located around Jinka town, approximately at 15 km to the North of Jinka. It has 50 small administrative units (kebeles) with a total population of 237,988. Among the 50 kebeles, four kebeles namely, Arkisha, Aykamer, Geza and Shepi were purposely selected for the present study based on the recent occurrence of Yf outbreak in the area. Bena-Tsemay district is found at 42 km to southeast of Jinka. The district has 32 kebeles with a total population of 74,853. Among the 32 kebeles, three kebeles namely, Luka, Goldia and Shaba-Argemenda were purposely selected for the present study based on the report of Yf outbreak occurrence. More detailed information on the study districts including a map showing the affected areas by the Yf outbreak has been described elsewhere [8].

### Study design, sample size estimation and data collection

Between March and May 2017, a community-based cross-sectional survey was conducted in the selected kebeles of the two districts. To our knowledge, there was no previous information on the level of community knowledge about Yf in the study area. Thus, assuming that 50% of adults will have high level of knowledge about Yf with 95% confidence in the estimate, 5% degree of accuracy, design effect of 1.5 and 90% response rate, a minimum sample size of 634 study participants would be required for the study.

Prior to data collection, a list of all the households in the selected kebeles was obtained from each of the respective kebele leaders. Based on the number of households in each of the selected kebeles, the pre-estimated sample size (634) was proportionally distributed. The required number of participants from each kebele was selected using systematic random sampling. The participants were eligible if they were residents of that kebele, age over 18 years, a husband/wife (or the responsible person) in the selected households, apparently healthy and volunteer to be interviewed.

Structured questionnaire was developed in English, based on information from available literatures regarding community knowledge, attitudes and practices about arboviruses from previous studies in other countries [13,14]. The questionnaire was translated into Amharic and pre-tested for clarity and acceptability in the study districts. During pre-testing, additional information was gathered and some of the questions were modified. The participants were interviewed about the public health importance, cause, mode of transmission, clinical symptoms, treatment, and preventive methods of Yf in their own local language (Ari, Bena and Tsemay) by trained health extension workers who were selected from each of the study kebeles. Each interview was made by a house–to-house visit. Information on the socio-demographic characteristics of the participants was also included in the questionnaire.

### Data management and analysis

The collected data were double-entered into a data entry file using EpiData software, V.3.1. The data were exported to Stata version 11 for statistical analysis. Pearson chi-square test was used to evaluate the statistically significant difference in the level of knowledge of signs and symptoms as well as sources of information about Yf, mode of transmission, practices of people and attitudes towards Yf between male and females. Bivariate and multivariable logistic regression analyses were performed to explore associations of socio-demographic characteristics of the study participants with increased odds of having higher levels of overall knowledge of Yf and to quantify the degrees of association using odds ratio. P-values below 5% were considered as indicators of statistical significance.

The overall knowledge of the study participants about Yf was assessed using the following eight main questions: (1) able to mention jaundice and/or vomiting blood as the severe sign/symptoms of Yf, (2) able to identify that Yf is different from malaria, (3) able to know that the treatment for Yf is different from malaria treatment, (4) able to know that Yf is transmitted from a patient to another person through mosquitoes bite, (5) able to mention that a mosquito which transmits malaria is different from a mosquitoes that transmit Yf, (6) able to mention that Yf can be transmitted from monkeys to human through mosquitoes bite, (7) able to mention that Yf is preventable by vaccine, and (8) able to know that Yf has a vaccine. Response to these questions were added together to generate overall knowledge score ranging from 0 to 8. A score of one was given to correct response, zero being used for incorrect/do not know response. Then, the response was categorized into a high,{those who scored 5 (60% cut-off point) and above} and a low,{score 4(50%) and below } overall knowledge of Yf as previously described [15].

Community’s practices regarding preventive methods of Yf were assessed by asking questions such as Yf is a preventable disease, preventive methods for Yf and are you/your family vaccinated for Yf (Table 4**)**. Gender difference in the proportion of answering correct response to each practice of question was evaluated using Pearson chi-square test. Similarly, study participants were asked questions such as “Yf is a public health problem”, “Yf is a newly occurred disease”, “Yf affects all age groups and Yf is a killer disease” (Table 5) to assess their attitudes. The association of gender and attitudes towards the disease was evaluated using Pearson chi-square test.

## Results

### Socio-demographic characteristics of the study participants

Table 1 shows the socio-demographic characteristics of the study participants. A total of 612 participants (55.9% males, age range from 18 to 87 years, mean age 33.36 years) participated in the study from the two areas, with a response rate of 96.5%. Among the study participants, 388 (63.4%) were recruited from the Debub Ari and the majority (65.2%) of the participants were illiterate.

**Table 1:** Socio-demographic characteristics of the study participants.

Characteristics	No. (%)
**Sex:**	
Male	342 (55.9)
Female	270 (44.1)
**Age group (year):**	
18–29	229 (37.4)
30–44	287 (46.9)
45–59	65 (10.6)
≥60	16 (2.6)
**Ethnicity**:	
Ari	381(62.3)
Bena Tsemay	220 (36.0)
Other	10 (1.6)
**Religion:**	
Protestant	286 (46.7)
Orthodox	92 (15.0)
Traditional	205 (31.0)
**Marital status:**	
Married	555 (90.7)
Single	14 (2.3)
Other	32 (5.2)
Educational status:	
Illiterate	399 (65.2)
Read only	72 (11.8)
Read and write	74 (12.1)
Primary (4–6)	26 (4.2)
Junior (7–8)	33 (5.4)
**Occupation:**	
Farmer	161(26.3)
Agro pastoralist	428 (69.9)
Other	23 (3.3)

### Community’s knowledge of signs/symptoms of Yf and sources of information

Table 2 shows communty’s knowledge of signs/symptoms of Yf and their sources of information. Out of the 612 study participants, 508 (83.0%) reported that they heard about Yf which is locally known as “a disease that causes vomiting blood”. The study participants reported that they heard about the disease from individuals who were sick from Yf (43.3%), followed from health workers (34.1%), friends (29.9%) and they have heard/seen a person who died of Yf (23.8%). A larger proportion of male participants had information about Yf compared to female participants (86.5% vs 78.5%, X^2^ = 6.89, p = 0.01). The most commonly mentioned signs and symptoms of the disease were vomiting blood (84.0%), fever (45%) and headache (43.8%). Regarding the cause of the disease, 112 (22.3%) responded that they knew its cause. However, only 8 (7.8%) mentioned a virus as the cause of Yf, while others mentioned bacteria/germ (42.2%) or other factors (50.0%).

**Table 2:** Community’s knowledge of signs & symptoms of Yf and sources of information.

Variables	Total No. (%)	Male No. (%)	Female No. (%)	, P Value
**Ever heard about Yf?**				, 0.01
Yes	508 (83.0)	296 (86.5)	212 (78.5)	
No	104 (17.0)	46 (13.5)	58 (21.5)	
**Source of information:**				
From sick person	217 (43.3)	126 (43.4)	91 (43.1)	0.94
Health worker	171 (34.1)	119 (41.0)	52 (24.6)	<0.01
Friend	150 (29.9)	84 (29.0)	66 (31.3)	0.58
Heard/seen person who died of Yf	119 (23.8)	63 (21.7)	56 (26.5)	0.211
Family member was sick	77 (15.4)	35 (12.1)	42 (19.9)	0.02
Media	14 (2.8)	12 (4.1)	2 (0.9)	0.03
**Do you know common symptoms of Yf?**				0.228
Yes	492 (97.2)	290 (98.0)	202 (96.2)	
No	14 (2.8)	6 (2.0)	8 (3.8)	
**Common symptoms of Yf:**				
Vomiting blood	411(84.0)	234 (81.0)	177 (88.5)	0.03
Fever	220 (45.0)	133 (46.0)	87 (43.5)	0.58
Headache	214 (43.8)	136 (47.1)	78 (39.0)	0.08
Jaundice	179 (36.6)	107 (37.0)	72 (36.0)	0.82
Nausea	162 (33.1)	93 (32.2)	69 (34.5)	0.59
Loss of appetite	87(17.8)	58 (20.1)	29 (14.5)	0.11
Bloody diarrhea	155 (31.7)	95 (32.9)	60 (30.0)	0.50
Other like weakness, muscle and joint pain	82(16.8)	53 (18.3)	29 (14.5)	0.92
**Yf is different from malaria**:				0.14
Yes	445 (90.5)	265 (91.4)	180 (89.1)	
No	12 (2.4)	9 (3.1)	3 (1.5)	
Do not know	35 (7.1)	16 (5.5)	19 (9.4)	
**The treatment is also different:**				0.07
Yes	358 (72.5)	222 (76.0)	136 (67.3)	
No	31 (6.3)	18 (6.2)	13 (6.4)	
Do not know	105 (21.3)	52 (17.8)	53 (26.2)	

Significantly higher proportion of participants from Debub Ari mentioned vomiting blood as the main symptoms of Yf compared to participants from Bena Tsemay (92.1% vs 69.0%, X^2^ = 44.39, P<0.001). Similarly, a significantly higher proportion of participants from Debub Ari reported jaundice as the symptom of Yf compared to those from Bena Tsemay (43.1% vs 24.6%, X^2^ = 16.44, P< 0.001). On the other hand, a high proportion of participants from Bena Tsemay area mentioned bloody diarrhea as the main symptom of Yf compared to those from Debub Ari (56.7% vs 18.2%, X^2^ = 76.08, p< 0.001). Majority (90.4%) of the study participants said that Yf is different from malaria and its treatment also different from malaria treatment (72.5%). Relatively, a higher proportion of males (76.0%) than females (67.3%) reported that the treatment for Yf is different from the treatment for malaria (X^2^ = 5.25, P = 0.07).

### Community’s knowledge regarding mode of transmission of Yf

Community’s knowledge regarding mode of transmission of Yf is summarized in Table 3. Less than half (41.9%) of the study participants said that Yf can be transmitted from a patient to another person. Among the 213 individuals who said that the disease can be transmitted, only 80 (37.6%) mentioned mosquitoes bite as a mode of transmission from person to person. More than half (55.9%) of the study participants thought that the disease can be transmitted from a patient to another person through breathing. A higher proportion of participants from Bena Tsemay mentioned mosquitoes bite as mode of transmission of the disease compared to those from Debub Ari (50.6% vs 28.2%, X^2^ = 11.02, p = 0.001). Few study participants also reported that they ever heard that this disease can be transmitted from monkeys to a person through mosquitoes bite or through drinking water contaminated with monkeys feces.

**Table 3:** Community’s knowledge regarding mode of transmission of Yf.

Variables	Total No. (%)	Male No. (%)	Female No. (%)	P value
**Yf can be transmitted from a patient to another person:**				0.115
Yes	213 (41.9)	135 (45.6)	78 (36.8)	
No	133 (26.2)	70 (23.7)	63 (29.7)	
Do not know	162 (31.9)	91 (30.7)	71 (33.5)	
**Mode of transmission**:				
Breathing	119 (55.9)	69 (51.1)	50 (64.1)	0.07
Mosquitoes bite	80 (37.6)	53 (39.3)	27 (34.6)	0.50
contact with a patient/body fluid	58 (27.2)	38 (25.2)	20 (20.4)	0.94
Sharing feeding material with a patient	27 (12.7)	18 (13.3)	9 (11.5)	0.70
Fly bites	8 (3.8)	4 (3.0)	4 (5.1)	0.42
**The same mosquitoes can transmit malaria and Yf:** Yes No Do not know	34 (42.5) 24 (30.0) 22 (27.5)	25 (47.2) 12 (22.6) 16 (30.2)	9 (33.3) 12 (44.4) 6 (22.2)	0.13
**Biting time of mosquitoes that transmits Yf:**				0.92
Night time	47 (61.0)	32 (62.8)	15 (57.7)	
Day time	4 (5.2)	3 (5.9)	1 (3.9)	
Both	16 (20.8)	10 (19.6)	6 (23.1)	
Do not know	10 (13.0)	6 (11.8)	4 (15.4)	
**Ever heard that Yf can be transmitted from monkeys to a person:**				0.96
Yes	19 (4.0)	11(3.9)	8 (4.0)	
No	462 (96.1)	270 (96.1)	192(96.0)	

### Community’s practices regarding preventive methods of Yf

Table 4 shows community’s practices regarding prevention of Yf as reported by the study participants. More than half (65.7%) of the participants thought that Yf is a preventable disease. Among those who believed that Yf is a preventable disease, majority (84.1%) mentioned vaccine as a preventive method. Most of the participants from Bena Tsemay believed that Yf is preventable through vaccine compared to those from Debub Ari (90.7% vs 79.9%, X^2^ = 6.88, P = 0.01). The participants mentioned stagnant water as the main breeding site for mosquitoes, and suggested avoiding stagnant water and use insecticide sprays as preventive methods of mosquitoes breeding. However, during the survey the research team did not ask the study participants /has not observed whether they practice avoiding stagnant water and use insecticide sprays as preventive methods of mosquitoes breeding.

**Table 4:** Community’s practices regarding prevention of Yf as reported by the study participants.

Variables	Total No. (%)	Male No. (%)	Female No. (%)	P value
**Yf is a preventable disease**:				0.43
Yes	333 (65.7)	193 (65.4)	140 (66.0)	
No	40 (7.9)	27 (9.2)	13 (6.1)	
Do not know	134 (26.4)	75 (25.4)	59 (27.8)	
**Preventive methods for Yf:**				
Vaccine	280 (84.1)	164 (85.0)	116 (82.9)	0.60
Preventing mosquitoes bite	53 (15.9)	29 (15.0)	24 (17.1)	
**Yf has a vaccine:**				0.06
Yes	318 (63.4)	190 (64.9)	128 (61.2)	
No	36 (7.2)	26 (8.9)	10 (4.8)	
Do not know	148 (29.5)	77 (26.3)	71 (34.0)	
**Are you/your family vaccinated for Yf:**				0.65
Yes	290 (91.5)	174 (92.1)	116 (90.6)	
No	27 (8.5)	15 (7.9)	12 (9.4)	
**Do you know a breeding site for mosquitoes?**				0.65
Yes	73 (91.3)	48 (92.3)	25 (89.3)	
No	7 (8.8)	4 (7.7)	3 (10.7)	
**Mosquitoes breeding sites:**				
Stagnant water	69 (94.5)	46 (95.8)	23 (92.0)	0.50
Marshy area	35 (47.9)	24 (50.0)	11 (44.0)	0.63
Axils of Enset plant	21 (28.8)	14 (29.2)	7 (28.0)	0.92
In discarded materials	21 (28.8)	14 (29.2)	7 (28.0)	0.92
Other like dirty place, rock	10 (13.7)	5 (10.4)	5 (20.0)	0.26

### Community’s attitudes about Yf

Table 5 shows attitudes of the study participants about the public health importance of Yf. Majority (86.2%) reported that Yf is a public health problem in their area, and 69.4% thought that Yf is a newly occurred disease in the area. A higher proportion (78.8%) of participants from Debub Ari considered Yf as a newly emerged disease compared to those from Bena Tsemay (52.8%) (X^2^ = 37.24, P< 0.001). Some of the participants suggested high rain fall (13.1%) and drought (4.5%) as the factors for the occurrence of the disease, while most (65.3%) of them had no idea about the factors contributing to its occurrence. The majority also believed that the disease affects all age groups (93.3%), it is a killer (97.2%) and not easily treatable (62.3%). A larger proportion (99.1%) of participants from Debub Ari said that Yf is a killer disease compared to those from Bena Tsemay (93.9%) (X^2^ = 13.04, P = 0.001). Almost all (99.4%) of the participants mentioned that if they suspect themselves or their families for Yf, they will visit health facility very soon.

**Table 5:** Community’s attitudes regarding the public health importance of Yf.

Variables	Total No. (%)	Male No. (%)	Female No. (%)	P value
**Yf is a public health problem**:				0.69
Yes	436 (86.2)	251 (85.4)	185 (87.3)	
No	28 (5.5)	16 (5.4)	12 (5.7)	
Not sure	42 (8.3)	27 (9.2)	15 (7.1)	
**Yf is a newly occurred disease:**				0.23
Yes	352 (69.4)	201(67.9)	151(71.7)	
No	141(27.8)	89 (30.1)	52 (24.6)	
Not sure	14 (2.8)	6 (2.0)	8 (3.8)	
**Yf affects all age groups**:				0.28
Yes	472 (93.3)	275 (93.2)	197 (93.4)	
No	14 (2.8)	6 (2.0)	8 (3.8)	
Not sure	20 (4.0)	14 (4.8)	6 (2.8)	
**Yf is a killer disease:**				0.60
Yes	492 (97.2)	285 (96.9)	207 (97.6)	
No	9 (1.8)	5 (1.7)	4 (1.9)	
Not sure	5 (1.0)	4 (1.4)	1 (0.5)	
**Do you fear that you /your family are at risk for Yf**?				0.87
Yes	450 (89.1)	263 (89.5)	187 (88.6)	
No	20 (4.0)	12 (4.1)	8 (3.8)	
Not sure	35 (6.9)	19 (6.5)	16 (7.6)	
**Yf is an easily treatable**:				0.67
Yes	137(27.1)	84 (28.6)	53 (25.0)	
No	315 (62.3)	179 (60.9)	136 (64.2)	
Not sure	54 (10.7)	31(10.5)	23 (10.9)	

### Community’s overall knowledge of Yf

Table 6 shows the overall knowledge of the study participants about Yf. Among the 612 study participants, 221(36.1%) were considered as having a high level of overall knowledge about Yf. Having Educational level of at least 7^th^ grade was significantly associated with a having high level of overall knowledge of the disease (COR = 2.6, 95%CI, 1.27, 5.34, P = 0.009 and AOR = 3.25, 95%CI, 1.39, 7.57, p = 0.006). Similarly, being resident of Bena-Tsemay district was associated with having a high level of overall knowledge of Yf compared to residents of Debub Ari (AOR = 1.77, 95% CI, 1.12, 2.78, P = 0.014). Agro-pastoralism as an occupation compared to farming was associated with having a low level of overall knowledge of Yf (COR = 0.65, 95% CI, 0.45, 0 .94, p = 0.022, and AOR = 0.51, 95% CI, 0.33, 0.79, P = 0.003).

**Table 6:** Association of socio-demographic characteristics of the study participants with overall knowledge of Yf.

Variables	Overall Knowledge		COR, (95% CI)	AOR, (95% CI)
	High No(%)	Low No (%)		
**Sex:**			Ref 0.83 (0.59,1.16)	Ref 0.92 (0.64,1.34)
Male	130 (38.0)	212 (62.0)		
Female	91 (33.7)	179 (66.3)		
**Age (year):**			Ref 1.17(0.82, 1.69) 1.16 (0.65, 2.05) 0.66 (0.21, 2.11)	Ref 1.37 (0.91, 2.06) 1.59 (0.84, 3.01) 0.78 (0.23, 2.66)
18–29	77 (33.6)	152 (66.4)		
30–44	107 (37.3)	180 (62.7)		
45–59	24 (36.9)	41 (63.1)		
≥60	4 (25.0)	12 (75.0)		
**Religion:**			Ref 1.54 (0.96, 2.48) 1.03 (0.71, 1.50)	Ref 1.50 (0.88, 2.55) 1.19 (0.75, 1.89)
Protestant	101 (35.3)	185 (64.7)		
Orthodox	42 (45.7)	50 (54.4)		
Tradition	74 (36.1)	131(63.9)		
**Education**				
Illiterate	126 (31.6)*	273 (68.4)	Ref	Ref
Read only	31 (43.1)	41 (56.9)	1.64 (0.98, 2.73)	1.74 (0.99, 3.06)
Read& write	32 (43.2)	42 (56.8)	1.65 (0.99, 2.74)	1.79 (1.01, 3.19)
Primary(4–6)	12 (46.2)	14 (53.9)	1.86 (0.83, 4.13)	1.98 (0.83, 4.74)
Junior (7–8)	18 (54.6)*	15 (45.5)	2.6 (1.27, 5.34)	3.25 (1.39,7.57)
**Occupation**				
Farmer	69 (42.9)	92 (57.1)	Ref	Ref
Agro Pastoralist	140 (32.7)*	288 (67.3)	0.65 (0.45, 0 .94)	0.51(0.33, 0.79)
Others	12 (52.2)*	11 (47.8)	1.45 (0.61, 3.49)	1.40 (0.48, 4.05)
**District**				
Debub Ari	137 (35.3)	251 (64.7)	Ref	Ref
Bena Tsemay	84 (37.5)	140 (62.5)	1.09 (0.78, 1.55)	1.77 (1.12, 2.78)

CI (Confidence Interval), COR (Crude odds ration), and AOR (Adjusted odds ratio)

## Discussion

In the present study area, Yf outbreaks have been repeatedly occurring since 1960 to the recent and caused thousands of deaths and cases [6–8].

Thus, it was assumed that at least 50% of the rural residents of the study area had a correct information about the cause, symptoms, mode of transmission and preventive methods of the disease. However, the results of this study indicated that most of community members (63.9%) had a low level of overall knowledge of the disease especially regarding its mode of transmission though the majority believed that the disease affects all age groups and it is a killer disease. The finding is in line with a previous study by Mohapatra and Aslami et al. [16] which revealed a low level of knowledge about Dengue fever among patients of a rural tertiary-care hospital in Sasaram, Bihar India, despite a good attitude. Comparable to the findings of this study, a community based study in Nepal [17], and study in India [14] also showed a low level of knowledge about Dengue fever despite the rapid spreads of the disease. In the current study, educational level above 7^th^ grade was found to be an indicator for a high level of overall knowledge of Yf which is similar to the findings of study elsewhere regarding knowledge of Dengue fever [13].

Among others, misconception about the symptoms, mode of transmission and preventive methods of a disease could highly affect individuals attitudes towards health-seeking behaviour and prevention. Infection with Yf virus shares non-specific clinical symptoms with other febrile illness, particularly with malaria [1, 18], which could affect timely care seeking behaviour of patients, and leading to delayed diagnosis/treatment in endemic areas due to limited diagnostic facilities. In the present study area, although the majority of the study participants could identify Yf as a disease which causes vomiting blood, there were community’s members who had no clear information regarding the difference between Yf and malaria (especially they confused it with falciparum malaria). Study in Tanzania also revealed that many community members believed that most instances of fever are due to malaria and the community had a low level of awareness about other non-malaria febrile illnesses like Rift Valley fever and Dengue fever despite the endemicity of the diseases [19]. In Ethiopia, Yf is known as *Bicha Woba* by health professionals. In Amharic language, *Bicha* means yellow, and *Woba* means malaria. On top of this, in Ethiopia there is no laboratory based effective diagnosis of Yf, and health professionals might not be fully aware of the magnitude of the disease.Thus the diagnosis may not be even considered, or may incorrectly be interpreted as malaria. The overall effects of these poor diagnosis practices in the health facilities and the name of the disease (*Bicha Woba)* could also be another factor which potentially affect community’s knowledge regarding the difference between Yf and malaria in the present study area. This necessitates creating community awareness on the common clinical features of Yf and malaria in the present study area where both diseases exist.

Studies suggest that understanding of local community’s knowledge regarding the mode of transmission of mosquitoes borne emerging and re-emerging viral diseases and their attitudes/practices towards prevention are among the essential elements for designing effective control measures [11, 20, 21]. The results of the present study indicate that majority of the community’s members had no correct information on the mode of transmission of Yf, as only 80 individuals mentioned that Yf can be transmitted from a patient to another person through mosquitoes bite, while only 24 individuals thought that mosquitoes which transmit Yf are different from those mosquitoes that transmit malaria. Contrary to our finding, a recent study in Angola showed that about 44% of the study participants had correct information regarding the transmission of Yf through mosquito bites [22]. Community’s knowledge regarding the transmission of the virus from non-human primates to human was also very poor. The results of study by Mohapatra and Aslami et al. [16] also showed that few patients had knowledge about *Aedes* mosquitoes as vectors of Dengue fever in Sasaram, Bihar India. The finding is also comparable to the findings of study on Rift Valley fever knowledge among agropastoral communities in Tanzania, where majority of the study participants had heard of the disease, but very few knew that mosquitoes can transmit the disease [15]. Providing information to community members through community health extension workers regarding the role of mosquitoes in the transmission of Yf and other diseases like malaria, Zika virus, Dengue fever etc, and teaching what to do to protect themselves/their families from mosquito bites in a simple understandable way would be helpful in increasing their awareness about major mosquito-borne diseases.

The results of this study also showed that the members of the community in the present study area regarded Yf as one of the most deadly diseases and most of the study participants acknowledged vaccination as the main preventive method. However, some of the study participants complained that though they and their families received a vaccine during the recent Yf outbreak in the area, they did not get adequate information about the specific vaccine/for what disease they are vaccinated, duration of protection of the vaccine and whether it is a vaccine or a treatment. This could affect the positive attitudes of individuals toward vaccine, and the health professionals who deliver vaccine could provide information on the specific vaccine and for what disease during vaccination campaigns, which in turn increases community’s attitudes and the desired vaccination coverage. Marlow et al., [22] also recommended the importance of providing clear information on Yf vaccination to the target population as some individuals did not understand whether the vaccine provided prevention or treatment in Angola where Yf outbreak was recently reported.

This study would provide information on the level of community’s knowledge, practices and attitudes about Yf in the studied area where such data was not available. However, the survey was conducted in two districts which were conveniently selected among the 6 districts of the South Omo Zone where cases and deaths were reported between 2012 and 2013. This is one of the limitations of the study which could affect the generalizability of the findings to all the different communities of the South Omo Zone. In addition, this quantitative study was not supplemented by qualitative study like focus group discussion which is important to gather detailed and additional information regarding community’s knowledge, practices and attitudes of Yf.

### Conclusion

Although Yf is becoming one of the most re-emerging mosquito-borne viral diseases in many African countries including the present study area, the findings of the present study showed that people living in endemic areas do not have adequate knowledge about its cause and mode of transmission though they consider it as one of the killer diseases. Although most of the study participants acknowledged vaccination as the main preventive method of Yf,some of the study participants complained that they did not get adequate information about the specific vaccine/for what disease they are vaccinated which affects the positive attitudes of individuals toward Yf vaccine. Thus, there is a need to increase people’s knowledge and practices regarding the cause, mode of transmission and preventive methods like avoiding mosquitoe breeding sites beside vaccination through various strategies like disseminating information through community health extension workers and community leaders.

## Supporting information

S1 Checklist STROBE checklist. (DOC)
